# Regulation of Nrf2/Keap1 signaling pathway in cancer drug resistance by galectin-1: cellular and molecular implications

**DOI:** 10.20517/cdr.2023.79

**Published:** 2024-02-29

**Authors:** İlhan Yaylim, Melek Aru, Ammad Ahmad Farooqi, Mehmet Tolgahan Hakan, Brigitta Buttari, Marzia Arese, Luciano Saso

**Affiliations:** ^1^Department of Molecular Medicine, Aziz Sancar Institute of Experimental Medicine, Istanbul University, Istanbul 34280, Turkiye.; ^2^Department of Medical Education, Istinye University Faculty of Medicine, Istanbul 34396, Turkiye.; ^3^Institute of Biomedical and Genetic Engineering (IBGE), Islamabad 54000, Pakistan.; ^4^Department of Cardiovascular and Endocrine-Metabolic Diseases, and Aging, Italian National Institute of Health, Rome 00161, Italy.; ^5^Department of Biochemical Sciences “A. Rossi Fanelli”, Sapienza University, Rome 00185, Italy.; ^6^Department of Physiology and Pharmacology “Vittorio Erspamer”, Sapienza University, Rome 00185, Italy.

**Keywords:** Galectin-1, Nrf2, oxidative stress, cancer drug resistance

## Abstract

Oxidative stress is characterized by the deregulation of the redox state in the cells, which plays a role in the initiation of various types of cancers. The activity of galectin-1 (Gal-1) depends on the cell redox state and the redox state of the microenvironment. Gal-1 expression has been related to many different tumor types, as it plays important roles in several processes involved in cancer progression, such as apoptosis, cell migration, adhesion, and immune response. The erythroid-2-related factor 2 (Nrf2)/Kelch-like ECH-associated protein 1 (Keap1) signaling pathway is a crucial mechanism involved in both cell survival and cell defense against oxidative stress. In this review, we delve into the cellular and molecular roles played by Gal-1 in the context of oxidative stress onset in cancer cells, particularly focusing on its involvement in activating the Nrf2/Keap1 signaling pathway. The emerging evidence concerning the anti-apoptotic effect of Gal-1, together with its ability to sustain the activation of the Nrf2 pathway in counteracting oxidative stress, supports the role of Gal-1 in the promotion of tumor cells proliferation, immuno-suppression, and anti-tumor drug resistance, thus highlighting that the inhibition of Gal-1 emerges as a potential strategy for the restraint and regression of tumor progression. Overall, a deeper understanding of the multi-functionality and disease-specific expression profiling of Gal-1 will be crucial for the design and development of novel Gal-1 inhibitors as anticancer agents. Excitingly, although it is still understudied, the ever-growing knowledge of the sophisticated interplay between Gal-1 and Nrf2/Keap1 will enable researchers to gain valuable insights into the underlying causes of carcinogenesis and metastasis.

## INTRODUCTION

The uncontrolled and irregular cellular proliferation, which represents a hallmark of cancer, requires an enormous amount of energy to accomplish extensive cell division^[1]^. Cells require an enormous amount of energy during division. The higher energy demand, with respect to normal cells, is associated with an increased level of reactive oxygen species (ROS) produced by transformed cells and released in the environment^[2]^. Chronic exposure to increased levels of ROS can cause DNA defects that may result in alteration of several enzymes’ activity and dysregulations in gene expression, thus leading to both structural and functional abnormalities within cells. However, the activation of specific cell defense mechanisms facilitates the efficient scavenging of reactive species and helps mitigate cell damage.

The role played by the nuclear factor erythroid-2-related factor 2 (Nrf2) is crucial in the prevention of oxidative stress-mediated cell damage. Nrf2 transcription factor activity is triggered in case of cell redox unbalance and oxidative stress establishment through its migration into the cell nucleus and the activation of more than 1,000 genes possessing an antioxidant response element (ARE) in their promoters^[3,4]^.

The normal functioning of the Nrf2 signaling is known as an important factor for brain homeostasis and repair following traumatic brain injury (TBI). Conversely, TBI disrupts the Nrf2 signaling pathway and triggers oxidative stress, inducing damage to the brain^[5]^.

The sensitivity of Nrf2 expression to high ROS levels in different cells involved in pregnancy is known. It has been reported that Nrf2, which helps maintain the redox balance in the cell during pregnancy, also decreases in the uterine tissues after birth^[6]^.

Studies have suggested that, apart from the normal role of Nrf2 during pregnancy, Nrf2 may also affect pathological mechanisms that lead to some disorders such as preeclampsia. Indeed, in mouse models, activated Nrf2 has been reported to impair placental angiogenesis, suppress fetal growth, and promote adverse maternal and neonatal outcomes^[7]^.

The molecular mechanisms involving cyclooxygenase-2 (COX-2), a key enzyme in prostaglandin biosynthesis, are not clear. Increased COX-2 expression during aging can affect antioxidative homeostasis as ROS production increases during acute and chronic inflammation, cellular senescence, and carcinogenesis^[8]^.

Moreover, COX-2 expression is dependent on Nrf2 under conditions of oxidative stress. It has been suggested that there is a synergistic relationship between COX-2 and Nrf2, which serves to regulate inflammation and contribute to an antioxidant response, ultimately yielding hepatoprotective effects^[9]^.

The regulatory activity of Nrf2 results in the expression of a wide number of cytoprotective proteins, including antioxidant, anti-inflammatory, and detoxifying enzymes, as well as proteins involved in the repair or removal of damaged macromolecules^[10-13]^. Nrf2 signaling suppresses pro-inflammatory cytokines and controls fundamental cellular processes such as apoptosis, autophagy, angiogenesis, proliferation, and cell cycle^[14]^.

Galectins, which have been shown in studies to be associated with oxidative stress and many cancer types, are proteins involved in the regulation of oxidative stress reactions and cellular differentiation^[15]^ and are small lectin molecules^[16]^ known as soluble β-galactosyl binding proteins^[17]^, which interact with both glycolipids and glycoproteins^[18]^. Some galectins require a reducing environment for the carbohydrate-binding reaction, a feature related to the presence of a different number of cysteine residues in their structure. For example, each monomer of galectin-1 (Gal-1) contains six cysteine (Cys) residues, and many studies have shown a critical relationship between the ligand binding effect of Gal-1 and the cysteine redox state^[19]^.

### GAL-1

Galectins are classified into three groups based on their carbohydrate recognition site (CRD) content and organization: (a) dimer-forming prototype galectins containing a single CRD, such as Gal-1, -2, -7, -10, -13 and -14; (b) consecutively containing two CRD regions such as Gal-4, -8, -9 and -12 (tandem repeats); (c) chimera galectin, the only example of this type of galectin is Gal-3^[20,21]^. Gal-1, which is encoded by the LGALS1 gene^[22]^ located in band 12 (22q12) of the long arm of chromosome 22 in humans, is known for its affinity for glycans containing β-galactoside^[23,24]^. Gal-1, approximately 29 kDa, has a noncovalent homodimer structure of single CRD subunits which can bind glycans both as a monomer and as a homodimer^[25,26]^.

### Gal-1 structure

The structure of human Gal-1 has a slightly bent bilayer β-sandwich-shaped three-dimensional structure with alternating long connecting rings^[27]^. Gal-1 in humans is a dimer in solution, and the structure of this dimer is protected by interactions of the monomers at the interface and by the hydrophobic core^[28]^. Gal-1 has a role in both acute and chronic inflammation by influencing immune cell adhesion, proliferation, apoptosis, and differentiation. It has been reported by *in-vivo* studies that extracellular Gal-1 can bind to glycan molecules expressed on the eosinophil cell surface in a carbohydrate-dependent manner, thereby inducing cell death by apoptosis and inhibiting cell migration^[29]^. A better understanding of the details related to the structural and functional properties of Gal-1 in the context of oxidative stress is crucial for the development of therapeutic approaches.

### Localization and modification of Gal-1

Gal-1 can be found in regions of the cell, such as the nucleus, cytoplasm, cell surface, and extracellular space. In addition to the keratinocyte cell surface in human skin, it has been found in both the cytoplasm and nuclei of Langerhans cells and fibroblasts^[30]^. Depending on its intracellular or extracellular localization, it can display different functions. Dimeric Gal-1 (dGal-1) serves extracellular functions, while monomeric Gal-1 operates independently of carbohydrates^[31]^. dGal-1 (subunit ~14.6 kDa) is a widely expressed dimeric protein. It is secreted by many cells, especially human endothelial cells^[32]^. It has been currently reported that Gal-1 had increased expression in many of the analyzed tumors, including skin cancer types, esophageal carcinoma, brain cancers, such as glioblastoma multiforme, diffuse large B-cell lymphoma of human head and neck, kidney carcinoma, sarcoma, gastric cancer, pancreatic cancer, and thymoma. However, no change or even a decline in Gal-1 expression has been found in ovarian cancer, cervical cancer, lung squamous cell carcinoma, or lung adenocarcinomas^[33]^. The secreted dGal-1 is found in the basement membrane and matrices around capillaries^[34]^. Oxidation of Gal-1 Cys triggers conformational changes that lead to the formation of three intramolecular disulfide bonds (Cys2-Cys130, Cys16-Cys88, and Cys42-Cys60), which prevent the carbohydrate-binding activity of Gal-1, thus causing the loss of its lectin function^[19]^. Gal-1 is also expressed by many immune cells, such as activated T and B cells^[35]^, macrophages^[36]^, regulatory T cells (Tregs cells)^[37]^, dendritic cells^[38]^. Many galectins have not signal sequence for secretion to extracellular space. However, some types of galectins such as Gal-1 is known to be in the extracellular part^[31,39]^.

Studies have shown that the detection of Gal-1 is an indication of tumor localization, depending on the tumor stage. Its expression is thought to be associated with a poor prognosis. A remarkable reduction in tumorigenesis was found in mice orthotopically implanted with Gal-1-silenced-MHCC97L cells. Of relevance, OTX008 (Gal-1 inhibitor) worked synergistically with sorafenib (multi-kinase inhibitor) in promoting the shrinkage of the tumors in mice inoculated with MHCC97L cells^[40]^.

#### Extracellular binding ligands of Gal-1

Depending on the different properties and numbers of glycoproteins on the cell surface, different cellular responses occur, mediated by Gal-1 binding to glycoproteins. Studies have shown that the extracellular proteins laminin and fibronectin do bind to Gal-1^[41]^. As in a receptor-ligand relationship, Gal-1 triggers signaling pathways within the cell upon binding to glycoproteins. Examples of glycoproteins that have a binding site with Gal-1 on the cell surface are integrins^[42,43]^, ganglioside-monosialic acid (GM1)^[44,45]^, CD146^[46]^, and neuro-pilin-1 (NRP-1). Extracellular Gal-1 binds specifically to the NRP1 protein by its CRD domain and induces phosphorylation of the vascular endothelial growth factor receptor 2, activation of the extracellular signal-amplified kinases 1/2 and of the c-Jun NH2-terminal kinase signaling cascades^[47]^.

#### Intracellular binding ligands of Gal-1

Gal-1 is involved in many intracellular signaling pathways independent of carbohydrate binding by interacting with proteins in the cytoplasm or nucleus^[48,49]^. In the nucleus, Gal-1 has been shown to form a complex with the Gemin4 protein, a component of the survival of motor neurons (SMN) core complex that plays a role in many biological processes^[50]^. Recent studies have shown that Gal-1 binds to the COOH terminal of active H-Ras, thereby stabilizing the plasma membrane. The interaction between H-Ras and Gal-1 is also important in tumor development and the stabilization of the H-Ras-guanosine triphosphate (GTP) complex at the cell membrane level^[51]^. Furthermore, intracellular Gal-1 can bind to H-Ras-GTP, the active form of H-Ras, independently of lactose. As a result of this interaction, H-Ras binds to the cell membrane and increases cell proliferation and migration^[49]^.

#### Gal-1 promotes immunosuppression in tumor microenvironment

Gal-1 has been shown to exert immunosuppressive functions in tumor microenvironment. Vaccination against Gal-1 significantly promoted the accumulation of CD3+ T cells and M1 macrophages in the tumor microenvironment. Importantly, considerable tumor suppression was found in subcutaneously vaccinated mice inoculated with melanoma cells^[52]^.

Gal-1-expressing breast cancer cells (4T1) were characterized by an increase in Treg cells, leading to immunosuppression and the initiation of lung metastases. Targeted inhibition of Gal-1 not only reduced the abundance of Treg cells but also impaired their suppressive capacity. Treg cells isolated from Gal-1-deficient tumors showed reduced suppressive activity compared to those obtained from the Gal-1-enriched tumor microenvironment^[53]^.

Gal-1 potently enhanced the establishment of premetastatic niches through myeloid-derived suppressor cells (MDSCs). (C-X-C motif) ligand 2 (CXCL2)/G protein-coupled chemokine receptor 2 (CXCR2) stimulates the migration of MDSCs in tumor-bearing mice. CXCR2 is present on the surface of MDSCs. High concentrations of CXCL2 efficiently promoted the chemotactic movements of MDSCs. Importantly, MDSCs created an immunosuppressive microenvironment and supported the metastatic dissemination of cancer cells. Gal-1 promoted the infiltration and accumulation of MDSCs in premetastatic niches through NFκB signaling. Mechanistically, Gal-1 triggered NFκB activation in tumor cells mainly through the stabilization of stimulator of interferon gene (*STING*), leading to prolonged inflammation-driven expansion of MDSCs^[54]^.

### OXIDATIVE STRESS ON NRF2/KELCH-LIKE ECH-ASSOCIATED PROTEIN 1 PATHWAY IN CANCER

The initiation of carcinogenesis, proliferation of cancer cells, spread of tumor cells, and progression of cancer as a result of oxidative stress-induced DNA mutation and damage show that oxidative stress is associated with cancer^[55]^. In this context, the regulation of oxidative stress has a very important place in both the evolution of the different cancer stages and in the development of anticancer treatments related to killing cancer cells. On the other hand, many signaling pathways associated with carcinogenesis directly or indirectly regulate the generation and metabolism of ROS. High levels of ROS under physiological conditions damage normal cells, whereas cancer cells produce and tolerate high levels of ROS. Due to the high energy demand associated with abnormal cell growth, tumor cells increase Adenosine triphosphate (ATP) generating processes, which leads to higher levels of ROS production^[56]^. Oncogenes increase the expression of Nrf2, which stimulates carcinogenesis by reducing the level of ROS to prevent the harmful effects of accumulated ROS products^[57]^. While Nrf2 protects the normal cell against carcinogens, it also protects the cancer cells from ROS and DNA damage and causes the spread of cancer^[58,59]^.

The presence of mutations of common oncogenes such as *BRAF*, *MYC*, and *KRAS* can increase NRF2 transcription and activity in malignant cells and save tumor cells from the ROS cytotoxicity caused by chemotherapeutic agents such as cisplatin and play a key role in drug resistance mechanisms^[60,61]^.

Although ABCF2 transporters are not considered transmembrane molecules because they do not have a transmembrane domain (TBD), unlike other ATP-binding cassette (ABC) transporters, studies have reported that they are overexpressed, especially in ovarian cancer cell lines. Additionally, cisplatin-resistant *ABCF2* gene amplification in cancer cell lines is suggested to play an important role in modulating cisplatin resistance. Bao *et al.* examined the role of ABCF2 in NRF2-mediated resistance to cisplatin in ovarian cancer cell lines. The ovarian cancer cell line overexpresses NRF2, which also exhibited elevated levels of ABCF2. It is demonstrated that there is increased resistance to cisplatin-induced apoptosis compared to its control counterpart. Conversely, the cell line with NRF2 knockdown and consequently lower levels of ABCF2 exhibited heightened sensitivity to cisplatin treatment compared to its control counterpart. Due to the presence of these data, it has been suggested that *ABCF2* may be a novel NRF2 target gene, playing an important role in cisplatin resistance in ovarian cancer^[62]^.

Although a significant portion of triple-negative breast cancer (TNBCs) initially respond to chemotherapeutic regimens, they often develop chemoresistance over time. Consequently, there is an urgent need to identify new molecular targets aimed at improving the treatment response in patients with TNBC post-chemotherapy. There are some studies focusing on the paraoxonase-2 (PON2) enzyme, which has demonstrated increased expression in some tumors, thereby contributing to disease chemoresistance and aggressiveness^[63]^.

Mechanisms by which PON2 contributes to resistance to cancer chemotherapy drugs have not yet been elucidated. It has been hypothesized that PON2-related chemoresistance activity exists. PON2 expression may lead to an anti-apoptotic effect due to increased ROS detoxification. Such anti-apoptotic roles of PON2 have been associated with the regulation of apoptosis caused by ER stress and mitochondrial superoxide anion production. A small number of studies have reported that decreased PON2 expression regulation is associated with increased intracellular ROS levels, which can damage DNA, proteins, and lipids^[64]^.

It has been reported that regulation to reduce PON2 can reduce cell proliferation in many types of cancer, including bladder cancer, pancreatic cancer, and highly aggressive brain cancers such as glioblastoma multiforme^[65-68]^.

The expression of Nrf2 is increased in many drug-resistant cancers^[69]^. Sferrazzo *et al.* observed that HO-1 overexpression was associated with drug resistance of brain tumors, such as astrocytoma, neuroblastoma, meningioma, and medulloblastoma^[70]^. Nrf2 triggered the expression of the *Bcl-xL* gene, which inhibits apoptosis and increases drug resistance in hepatocellular cancer cells (Hepa-1 and HepG2)^[71]^.

### The Nrf2 structure and regulation

**Figure 1:**
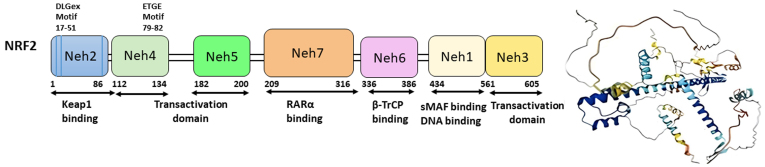
Functional domains (Neh1-Neh7) and crystal structures of Nrf2 (Figure adapted from^[72]^). Nrf2: Erythroid-2-related factor 2.

Nrf2, which is encoded by the *NFE2L2* gene, belongs to the Cap’n’collar (CNC) transcription factor family^[14]^. It comprises 605 amino acids and is divided into seven functional domains (Neh1-Neh7) [Figure 1].

**Figure 2:**
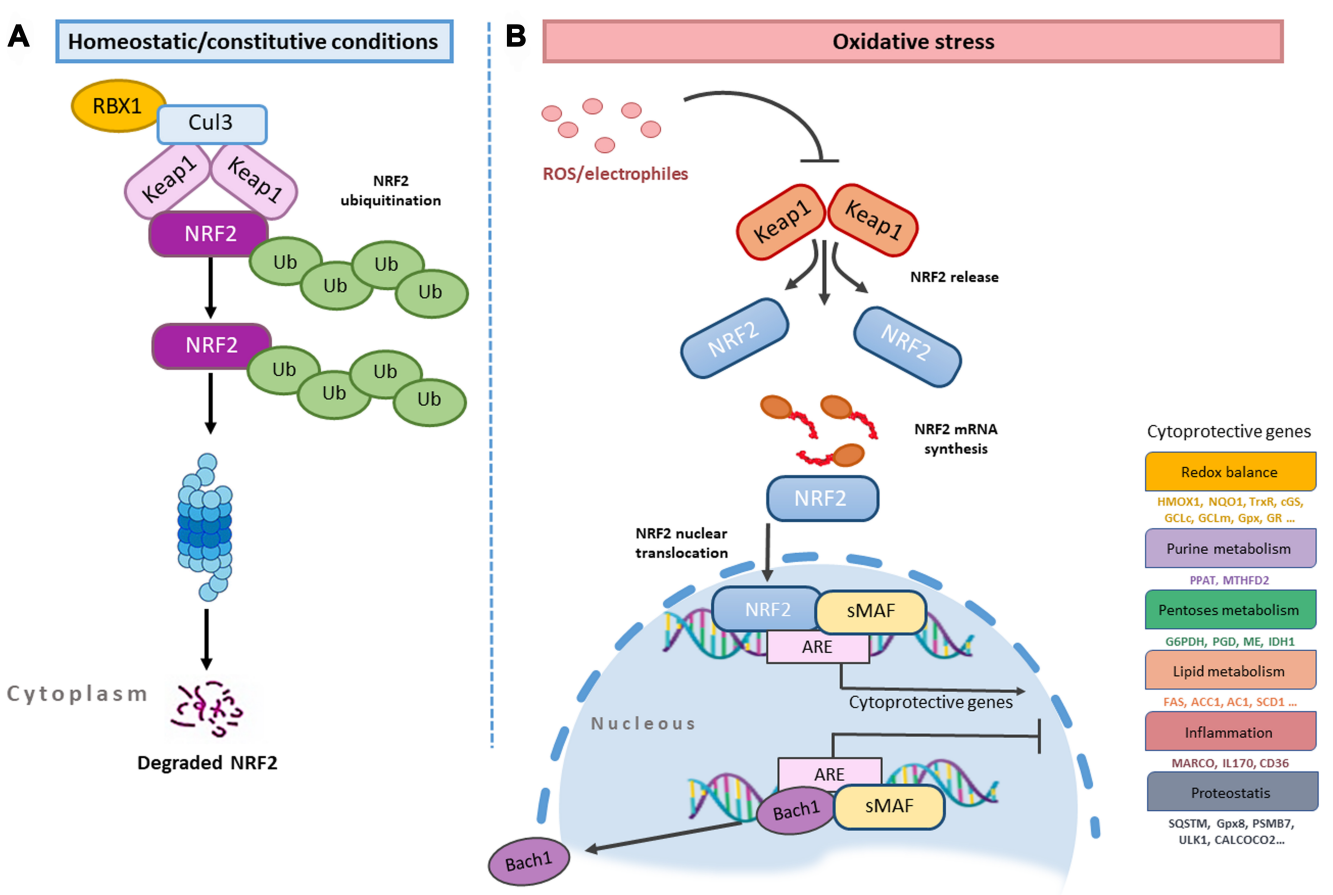
Nrf2/Keap1 signaling pathway. (A) homeostatic/constitutive conditions; (B) oxidative stress conditions (Figures adapted from^[73-84]^). Nrf2: Erythroid-2-related factor 2; Keap1: Kelch-like ECH-associated protein 1.

Kelch-like ECH-associated protein 1 (Keap1), the main negative regulator of Nrf2, provides both the ubiquitination and stability of Nrf^[73]^. In the absence of stress, Nrf2 in the cytoplasm is held by the Keap1/Cullin 3 (CUL3)/RBX1 E3-ubiquitin ligase complex [Figure 2A] and keeps the expression of ARE-responsive genes at a basic level due to following proteasomal degradation^[74]^. The concentration of the Keap1 protein varies with cell type, and the rate of ubiquitination and degradation of Nrf2 depends on it in non-stressed cells^[75]^. When the cell is exposed to stress, which may be due to exogenous and endogenous factors, the Nrf2/Keap1/ARE signaling pathway is activated^[76,77]^. Electrophilic inducers reacting with Keap1 at the level of sensitive Cys residues^[78]^ induce a change in the Keap1-Nrf2 protein-protein interaction, causing Nrf2 to be synthesized and subsequently translocated into the nucleus^[79,80]^, where it dimerizes with the small MAF (sMAF) protein to form the Nrf2-sMAF complex^[81-84]^ [Figure 2B].

The formation of the Nrf2-sMAF complex ensures the induction of phase II detoxifying enzyme genes with Nrf2 binding to ARE of various genes. Examples of these genes are heme-oxygenase-1 (HMOX1), glutamate-cysteine ligase catalytic (GCLC) and modifier subunit (GCLM), UDP-glucuronosyltransferase (UGT), NAD(P)H quinone oxidoreductase 1 (NQO1), sulfiredoxin1 (SRXN1), glutathione S-transferase (GST), and multidrug resistance-associated proteins (MRPs)^[85-88]^. In homeostatic conditions, MAF proteins and BACH1 (BTB and CNC homology 1) form heterodimers and they occupy AREs. BACH1, the sensor of heme molecule and natural competitor of Nrf2 in the cell, also acts as a transcription repressor^[89]^. If the cell receives a stress signal, BACH1 is phosphorylated at Tyr 486 and comes out of the nucleus, thus allowing the Nrf2 free access to the ARE^[90]^. When the redox balance is stabilized by Nrf2, the resynthesized BACH1 enters the nucleus and represses the ARE elements. Oxidative stress is often associated with heme release from hemoproteins. As the free heme level rises, the binding of BACH1 and FBXO22 ubiquitin ligase increases, thereby addressing BACH1 to proteasome degradation^[91]^. This model has been extensively investigated by programming the experimental model with the genetic background to HMOX1.

Lignitto *et al.* have compared genetically programmed KP (Kras^LSL-G12D/+^; p53^flox/flox^) mice with KPK mice and have observed KPK (Keap1 knockout) mice to develop a high number of lung metastases dependent on both Nrf2 and Bach1. The mechanisms that reduce the level of BACH1 degradation are as follows: (a) Increased heme oxygenase 1 level depending on Nrf2; (b) Increased heme degradation rate; (c) Decreased FBXO22 ubiquitin ligase and BACH1 depending on the concentration of free heme; (d) Increased levels of free heme effects proteasome-dependent BACH1 breakdown. With this study, researchers have indicated that the deficiency of FBXO22 has highly effectively activated the premetastatic transcription program of BACH1 in KPK cells. In this complicated relationship, the promoter activity of BACH1 was demonstrated depending on its multiple interactions; thus, while HMOX1 was shown to exert an inhibitory activity in the progress of metastasis, BACH1 plays important roles in the poor survival and formation of metastasis in lung adenocarcinoma^[92]^.

In agreement with the reported study, further evidence indicates a relationship between increased BACH1 level and metastasis, observed by lowering the level of free antioxidants such as vitamin E and NAC (N-acetyl cysteine). It has also been shown that Bach1 contributed to the energy production (ATP) and glycolysis enhancement by binding to Glyceraldehyde-3-Phosphate Dehydrogenase (GAPDH) and Hexokinase 2 (Hex 2)^[93]^.

All these findings suggest that the Nrf2 signaling is linked by a complex relationship to other pathways. In addition, Nrf2-targeted therapy methods should be explored by examining the roles of Nrf2 in various cancers in more detail^[94]^.

### The dual role of Nrf2 in tumorigenesis

As a transcription factor, Nrf2 is considered a master regulator of the cellular antioxidant response. Although its activity was initially assumed to prevent cancer and disease, recent studies have shown that the Nrf2 signaling pathway also plays a role in cancer initiation, spread, and resistance to treatment^[95]^. In physiological conditions, a low Nrf2 level in the nucleus is desired to sustain the homeostasis of the cell. Low levels of Nrf2 reduce the effects of factors such as ROS, carcinogens, and chemicals that cause DNA damage. Low levels of Nrf2 diminish the body’s ability to counteract factors such as ROS, carcinogens, and chemicals that can induce DNA damage, thereby leading to tumor initiation and increased metastasis^[96]^. The escalation of DNA damage, coupled with the inability to repair it, causes the resulting cancer cells to produce high amounts of ROS and activate Nrf2. Nrf2 activation, on the other hand, enables cancer cells to evade apoptosis and thus prompts the expression of genes that facilitate their proliferation. Studies have shown that cancers with high levels of Nrf2 are linked to a poor prognosis. As a result, while the activation of the Nrf2 signaling pathway offers protective effects in the early stages of carcinogenesis, it becomes undesirable in the later stages. Thus, while elevating Nrf2 is crucial for cancer prevention, reducing its levels is imperative for treatment^[97,98]^.

## GAL-1 PROMOTES TUMOR PROGRESSION BY T-CELL EXHAUSTION

Gal-1 plays a role in many stages of carcinogenesis processes. In the invasion stage, together with integrins, it adheres to proteins in the ECM and facilitates tumor cell adhesion. Gal-1, which facilitates the adhesion of the tumor cell, also ensures the separation of the cancer cell for metastasis to occur. It also helps the next process, i.e., the escape of cancer cells from the immune system. The mechanism of T cells killing cancer cells occurs in two ways, including secretion of perforin and granzyme enzymes, and exocytosis. Cancer cells can evade immune responses by secreting immunosuppressive cytokines and inhibitory factors such as Gal-1. Gal-1 promotes the escape of cancer cells from T cells by increasing apoptosis^[99,100]^. Gal-1-overexpressing pancreatic stellate cells significantly induced apoptotic death in CD4+ and CD8+ T cells^[101]^.

Gal-1-targeting DNA aptamers (AP-74 M-545) have been reported to be effective against lung cancer models. Intratumoral injections of AP-74 M-545 inhibited tumor growth in C57BL/6 mice injected with LL/2 cells. Essentially, binding of Gal-1 to CD45 on T cells caused activation of apoptotic signaling in T-cells. AP-74 M-545 also potently promoted the infiltration and accumulation of CD4+ and CD8+ T cells in tumor tissues^[102]^. There is sufficient proof-of-concept related to the Gal-1-mediated inactivation of immune cells^[103]^.

There is an exciting piece of evidence suggesting that increased infiltration of interferon gamma (IFNγ)-producing activated T cells in the tumor tissues also triggers the upregulation of PD-L1 in the cancer cells. B-Raf mutations in melanoma cells make them therapeutically resistant. Vemurafenib targets B-Raf and exerts suppressive effects on Programmed Cell Death Protein 1 (PD-L1) levels mainly through inhibition of signal transducer and activator of transcription 3 (STAT3)-mediated upregulation of PD-L1. However, vemurafenib also stimulates the expression of oncogenic Gal-1 in melanoma cells. Gal-1 expressing-A375 and MEL28 melanoma cells induced apoptosis in T cells. Therefore, vemurafenib efficiency can be improved principally through inhibition of Gal-1 in melanoma cells^[104]^.

Overall, Gal-1-mediated exhaustion of T cells^[105,106]^ is a rapidly growing concern and interdisciplinary researchers are focusing on the pharmacological targeting of Gal-1 to enhance the functionalities of T cells.

Gal-1 has also been reported to exert anti-apoptotic effects in cervical and lung cancers^[107,108]^.

Importantly, because of the rapid pace of phenomenal advancements in glycobiology, pharmacological targeting of Gal-1 has generated compelling evidence related to inhibition of carcinogenesis and metastasis.

## GAL-1 AND DRUG RESISTANCE

Gal-1, overexpressed in hepatocellular carcinoma cells (HepG2), increases the expression of P-glycoprotein (P-gp), causing drug resistance against doxorubicin and sorafenib^[109]^. In addition, Gal-1 induces drug resistance by inducing epithelial-mesenchymal transition (EMT) in hepatocellular carcinoma cells^[110]^. Gal-1 overexpressed in patients with triple-negative breast cancer caused the development of doxorubicin resistance through interaction with integrin β1, which activates FAK/c-Src/ERK/STAT3 signaling. In the same study, inhibition of Gal-1 was also shown to increase doxorubicin-induced apoptosis in human breast cancer cells^[111]^. Wang *et al.* demonstrated in a study of patients with breast cancer that the degradation of Gal-1 improved the drug sensitivity of breast cancer by reducing the expression of P-gp by inhibiting the Raf-1/AP-1 pathway^[112]^. Apart from oxidative stress, Gal-1 also regulates endoplasmic reticulum (ER) stress. Targeted inhibition of Gal-1 in Hs683 orthotopic xenografts in mouse brains impaired ER stress responses and improved the therapeutic efficacy of the drug temozolomide^[113]^.

The anti-tumoral effect of thiodigalactoside has been described in murine cancer, elicited by the inhibition of Gal-1-mediated protection against oxidative stress. This evidence supports the role of Gal-1 in lowering oxidative stress and inhibiting apoptosis and indicates Gal-1 inhibition as a condition required to trigger oxidative stress-mediated apoptosis^[114]^. In terms of antioxidant status, Nrf2 can upregulate genes that have a role in thioredoxin usage and regeneration, which may affect glutathione levels^[115]^. Raffaghello *et al.* proposed a hypothesis regarding short-term starvation (STS) or low serum/low glucose, suggesting that they may offer protection to normal mammalian cells againts increased oxidative damage or chemotherapeutic drugs, but not, or to a lesser extent, to cancer cells^[116]^.

Pateras *et al.* conducted an *in-vitro* study about the molecular mechanism of fasting or STS. Their primary aim was to elucidate the unclear mechanism behind the increased ROS production in malign breast cancer cell lines exposed to STS+ doxorubicin (DXR). They determined the transcriptional activity of Nrf2 and two important antioxidant target genes thioredoxin reductase 1 (*TXNRD1*) and NAN(P)H quinone dehydrogenase (*NQO1*). The study concluded that the combined STS+DXR treatments in triple-negative breast cancer cell lines were associated with a reduction in messenger RNA levels of TXNRD1 and NQO1, which are transcriptional targets of Nrf2, along with increased ROS levels^[117]^.

## OXIDATIVE STRESS AND GALECTINS

Oxidative stress is an important factor contributing to the imbalance between increased ROS and the activation of antioxidant pathways, thereby leading to the occurrence of many diseases. Current research highlights the important role of galectins in processes related to the onset and progression of diseases through various mechanisms mediated by impaired cell redox homeostasis and ROS-mediated oxidative stress^[118,119]^.

Several galectin types can directly stimulate a respiratory burst in phagocyte cells by inducing NADPH oxidase, which produces superoxide and hydrogen peroxide. It is also known that the galectin function is associated with redox state, environmental status, and oxidative stress dependence due to the altering MAPK, MEK1/ERK, mTOR, and JNK signaling pathways^[36,120-125]^.

The relationship of Gal-1 with oxidative stress has been reported, as proved by the increased production of reactive oxygen species in the cell, enabling myofibroblast activation and also affecting the migration of these cells. NADPH oxidase 4 (NOX4), which enables the production of reactive oxygen species, is upregulated by Gal-1 through the neuropilin-1/Smad3 signaling pathway in myofibroblast cells^[15]^.

T cell immunoglobulin and mucin domain-containing protein 3 (TIM3) has a central role in different molecular mechanisms^[126,127]^. Galectin 9 has two carbohydrate recognition domains and can effectively trigger the oligomerization of TIM3. TIM3/Gal-9 interactions protect acute myeloid leukemia cells from oxidative stress^[128]^.

## INTERPLAY BETWEEN GAL-1 AND NRF2 SIGNALING PATHWAY

Several pieces of evidence link the effect of Gal-1 with Nrf2 activation, and outline the impact of this signal in the regulation of inflammation and apoptosis^[129]^.

Gal-1 has the ability to protect neurons from cellular stress by preventing apoptosis and reducing the levels of ROS. Essentially, the neuroprotective effects of Gal-1 were found to be abolished upon the knockdown of Nrf2^[130]^.

To investigate whether autophagy is associated with the AMPK signaling pathway when treated with oxaliplatin, analyses were performed in colon cancer (CRC) cells, and it was shown that oxaliplatin resistance is regulated by the AMPK signaling pathway^[131]^. Further research is needed to develop specific inhibitors for AMPK that can be used in cancer treatment. AMPK and Nrf2 are both affected and activated by stress in the cell, and when activated, they regulate the cellular balance^[132,133]^.

A study showed that Gal-1 has protective effects in lipopolysaccharide (LPS)-induced Acute lung injury (ALI). Treatment of LPS-induced ALI mice models with Gal-1 reduced LPS-induced lung tissue damage and increased tissue healing by protecting against oxidative stress. Gal-1 inhibited TXNIP-NLRP3 inflammatory activation in LPS-treated macrophages by directly binding to NLRP3 protein. It also attenuated LPS-related lung injury by activation of NRF-2 in association with AMPK phosphorylation. Gal-1 reduced LPS-mediated pro-inflammatory cytokines and ROS generation while increasing antioxidative enzymes regulated by the AMPK/Nrf2 pathway in primary macrophages. Gal-1 upregulates Nrf2 by activating the AMPK signaling pathway, thus showing a protective effect against LPS-related ALI as a result of inhibition of oxidative stress. In the abovementioned study, Gal-1 was suggested as an effective agent in the treatment of ALI^[134]^.

## GAL-1 AND NRF2 AS A POTENTIAL THERAPEUTIC TARGET IN CANCER AND DRUG RESISTANCE

Gal-1 has promising therapeutic potential for diseases caused by inflammation and also shows promise in cancer therapy^[100,135,136]^. Small galectin inhibitor molecules, such as OTX008, have been used to treat galectin-induced tumorigenesis. OTX008 has been shown to bind to the CRD of Gal-1 and inhibit tumor cell survival and angiogenesis in ovarian cancer^[137,138]^. The small molecule 6DBF7 is a dibenzofuran (DBF)-based peptidomimetic of Gal-1, whose galectin inhibitor activity was shown as it inhibited tumor angiogenesis and tumor growth in mouse cancer models such as melanoma, lung, and ovarian cancer^[137]^. It was shown that an anti-Gal-1 (F8.G7) monoclonal antibody inhibited tumor angiogenesis and increased tumor regression in a mouse model of Kaposi’s sarcoma^[138]^. Further evidence suggested that Nrf2 may have a dual role in cancer, as it protects normal cells against carcinogenesis and induces the expression of genes involved in the survival and proliferation of cancer cells. Since there is concern about the cytotoxicity of Nrf2 activators used in therapy, more research should be conducted on the specificity and mechanism of action of Nrf2 inhibitors^[15]^.

## FUTURE PERSPECTIVES AND CONCLUSIONS

It is very important to achieve a cancer chemopreventive effect to minimize the risk of cancer development by applying natural or synthetic chemical agents. One of the most suitable and widely explained approaches for this is to provide the induction of enzymes that have detoxifying and cytoprotective effects. In this case, the Nrf2/Keap1 signaling pathway plays a critical role in regulating the expression of genes encoding many inducible enzymes. To date, studies continue on many natural Nrf2 activators, semisynthetic and synthetic Nrf2 activators, natural Nrf2 inhibitors, semisynthetic and synthetic Nrf2 inhibitors, which affect the Nrf2/Keap1 signaling pathway and thus have the ability to regulate genes involved in antioxidant defense.

The mechanisms by which Nrf2 and Gal-1 regulate free radical levels in cellular with Nrf2 activation remain unclear. For the treatment of cancers associated with the Nrf2 signaling pathway, the development of specific inhibitors of Nrf2 and Gal-1 is necessary. As the role of Gal-1 on tumor onset and spread is investigated, it is predicted that Gal-1 inhibitors will cause delays in tumor progression and prolong survival. A strong effort should be made to minimize the side effects eventually exerted by Gal-1 inhibitors intended for use in the treatment of cancer patients. Scientists are increasingly approaching new therapeutic developments to unveil the effective role played by Gal-1 at the molecular level and to understand its effects on the cancer process. Inhibition of Gal-1 expression and NRF-2 regulation should be studied for treatments for various types. The differentiation of stress-sensitive and resistant galectins against oxidative stress is still not clear, and once the mechanism of action of the specific galectin subtype is available, it will allow it to counteract ROS-induced cell damage and address more focused anticancer treatments. Together with the observations of opposite effects of galectin inhibitors on cellular differentiation and proliferation, these findings highlight the importance of individual galectin approaches for developing anticancer and anti-inflammatory therapeutics.

## DECLARATIONS

### Copyright

© The Author(s) 2024.

## References

[1] Hassanpour SH, Dehghani M (2017). Review of cancer from perspective of molecular. J Cancer Res Pract.

[2] Chio IIC, Jafarnejad SM, Ponz-Sarvise M (2016). NRF2 promotes tumor maintenance by modulating mRNA translation in pancreatic cancer. Cell.

[3] Malhotra D, Portales-Casamar E, Singh A (2010). Global mapping of binding sites for Nrf2 identifies novel targets in cell survival response through ChIP-Seq profiling and network analysis. Nucleic Acids Res.

[4] Tonelli C, Chio IIC, Tuveson DA (2018). Transcriptional regulation by Nrf2. Antioxid Redox Signal.

[5] Abdul-Muneer PM (2023). Nrf2 as a potential therapeutic target for traumatic brain injury. J Integr Neurosci.

[6] Lu J, Wang Z, Cao J, Chen Y, Dong Y (2018). A novel and compact review on the role of oxidative stress in female reproduction. Reprod Biol Endocrinol.

[7] Nezu M, Souma T, Yu L (2017). Nrf2 inactivation enhances placental angiogenesis in a preeclampsia mouse model and improves maternal and fetal outcomes. Sci Signal.

[8] Zdanov S, Toussaint O, Debacq-Chainiaux F (2009). p53 and ATF-2 partly mediate the overexpression of COX-2 in H_2_O_2_-induced premature senescence of human fibroblasts. Biogerontology.

[9] Fuertes-Agudo M, Luque-Tévar M, Cucarella C, Martín-Sanz P, Casado M (2023). Advances in understanding the role of NRF2 in liver pathophysiology and its relationship with hepatic-specific cyclooxygenase-2 expression. Antioxidants.

[10] Dinkova-Kostova AT, Abramov AY (2015). The emerging role of Nrf2 in mitochondrial function. Free Radic Biol Med.

[11] Ma Q (2013). Role of nrf2 in oxidative stress and toxicity. Annu Rev Pharmacol Toxicol.

[12] Chen B, Lu Y, Chen Y, Cheng J (2015). The role of Nrf2 in oxidative stress-induced endothelial injuries. J Endocrinol.

[13] Negi CK, Jena G (2019). Nrf2, a novel molecular target to reduce type 1 diabetes associated secondary complications: the basic considerations. Eur J Pharmacol.

[14] Khassafi N, Azami Tameh A, Mirzaei H (2024). Crosstalk between Nrf2 signaling pathway and inflammation in ischemic stroke: mechanisms of action and therapeutic implications. Exp Neurol.

[15] Vinnai JR, Cumming RC, Thompson GJ, Timoshenko AV (2017). The association between oxidative stress-induced galectins and differentiation of human promyelocytic HL-60 cells. Exp Cell Res.

[16] Kasai K, Hirabayashi J (1996). Galectins: a family of animal lectins that decipher glycocodes. J Biochem.

[17] Levi G, Teichberg VI (1981). Isolation and physicochemical characterization of electrolectin, a beta-D-galactoside binding lectin from the electric organ of electrophorus electricus. J Biol Chem.

[18] Coppin L, Jannin A, Ait Yahya E (2020). Galectin-3 modulates epithelial cell adaptation to stress at the ER-mitochondria interface. Cell Death Dis.

[19] Guardia CM, Caramelo JJ, Trujillo M (2014). Structural basis of redox-dependent modulation of galectin-1 dynamics and function. Glycobiology.

[20] Liu FT, Rabinovich GA (2010). Galectins: regulators of acute and chronic inflammation. Ann N Y Acad Sci.

[21] Di Lella S, Sundblad V, Cerliani JP (2011). When galectins recognize glycans: from biochemistry to physiology and back again. Biochemistry.

[22] Camby I, Le Mercier M, Lefranc F, Kiss R (2006). Galectin-1: a small protein with major functions. Glycobiology.

[23] Chetry M, Thapa S, Hu X (2018). The role of galectins in tumor progression, treatment and prognosis of gynecological cancers. J Cancer.

[24] Zhu J, Zheng Y, Zhang H, Liu Y, Sun H, Zhang P (2019). Galectin-1 induces metastasis and epithelial-mesenchymal transition (EMT) in human ovarian cancer cells via activation of the MAPK JNK/p38 signalling pathway. Am J Transl Res.

[25] Barondes SH, Castronovo V, Cooper DN (1994). Galectins: a family of animal beta-galactoside-binding lectins. Cell.

[26] Cho M, Cummings RD (1995). Galectin-1, a beta-galactoside-binding lectin in Chinese hamster ovary cells. I. Physical and chemical characterization. J Biol Chem.

[27] Tracey B, Feizi T, Abbott W, Carruthers R, Green B, Lawson A (1992). Subunit molecular mass assignment of 14,654 Da to the soluble beta-galactoside-binding lectin from bovine heart muscle and demonstration of intramolecular disulfide bonding associated with oxidative inactivation. J Biol Chem.

[28] López-Lucendo MF, Solís D, André S (2004). Growth-regulatory human galectin-1: crystallographic characterisation of the structural changes induced by single-site mutations and their impact on the thermodynamics of ligand binding. J Mol Biol.

[29] Ge XN, Ha SG, Greenberg YG (2016). Regulation of eosinophilia and allergic airway inflammation by the glycan-binding protein galectin-1. Proc Natl Acad Sci U S A.

[30] Rabinovich GA, Toscano MA (2009). Turning ‘sweet’ on immunity: galectin-glycan interactions in immune tolerance and inflammation. Nat Rev Immunol.

[31] Perillo NL, Marcus ME, Baum LG (1998). Galectins: versatile modulators of cell adhesion, cell proliferation, and cell death. J Mol Med.

[32] Hafer-Macko C, Pang M, Seilhamer JJ, Baum LG (1996). Galectin-1 is expressed by thymic epithelial cells in myasthenia gravis. Glycoconj J.

[33] Rudjord-Levann AM, Ye Z, Hafkenscheid L (2023). Galectin-1 induces a tumor-associated macrophage phenotype and upregulates indoleamine 2,3-dioxygenase-1. iScience.

[34] Wasano K, Hirakawa Y, Yamamoto T (1990). Immunohistochemical localization of 14 kDa beta-galactoside-binding lectin in various organs of rat. Cell Tissue Res.

[35] Akazawa C, Nakamura Y, Sango K, Horie H, Kohsaka S (2004). Distribution of the galectin-1 mRNA in the rat nervous system: its transient upregulation in rat facial motor neurons after facial nerve axotomy. Neuroscience.

[36] Almkvist J, Karlsson A (2002). Galectins as inflammatory mediators. Glycoconj J.

[37] Rabinovich GA, Ariel A, Hershkoviz R, Hirabayashi J, Kasai KI, Lider O (1999). Specific inhibition of T-cell adhesion to extracellular matrix and proinflammatory cytokine secretion by human recombinant galectin-1. Immunology.

[38] (2015). de Freitas Zanon C, Sonehara NM, Girol AP, Gil CD, Oliani SM. Protective effects of the galectin-1 protein on in vivo and in vitro models of ocular inflammation. Mol Vis.

[39] Orozco CA, Martinez-Bosch N, Guerrero PE (2018). Targeting galectin-1 inhibits pancreatic cancer progression by modulating tumor-stroma crosstalk. Proc Natl Acad Sci U S A.

[40] Leung Z, Ko FCF, Tey SK (2019). Galectin-1 promotes hepatocellular carcinoma and the combined therapeutic effect of OTX008 galectin-1 inhibitor and sorafenib in tumor cells. J Exp Clin Cancer Res.

[41] Kopitz J, von Reitzenstein C, Burchert M, Cantz M, Gabius HJ (1998). Galectin-1 is a major receptor for ganglioside GM1, a product of the growth-controlling activity of a cell surface ganglioside sialidase, on human neuroblastoma cells in culture. J Biol Chem.

[42] Tinari N, Kuwabara I, Huflejt ME, Shen PF, Iacobelli S, Liu F (2001). Glycoprotein 90K/MAC-2BP interacts with galectin-1 and mediates galectin-1-induced cell aggregation. Int J Cancer.

[43] Thijssen VL, Barkan B, Shoji H (2010). Tumor cells secrete galectin-1 to enhance endothelial cell activity. Cancer Res.

[44] Jung TY, Jung S, Ryu HH (2008). Role of galectin-1 in migration and invasion of human glioblastoma multiforme cell lines. J Neurosurg.

[45] Wu MH, Hong TM, Cheng HW (2009). Galectin-1-mediated tumor invasion and metastasis, up-regulated matrix metalloproteinase expression, and reorganized actin cytoskeletons. Mol Cancer Res.

[46] Camby I, Belot N, Lefranc F (2002). Galectin-1 modulates human glioblastoma cell migration into the brain through modifications to the actin cytoskeleton and levels of expression of small GTPases. J Neuropathol Exp Neurol.

[47] Wu MH, Ying NW, Hong TM, Chiang WF, Lin YT, Chen YL (2014). Galectin-1 induces vascular permeability through the neuropilin-1/vascular endothelial growth factor receptor-1 complex. Angiogenesis.

[48] Patterson RJ, Wang W, Wang JL (2002). Understanding the biochemical activities of galectin-1 and galectin-3 in the nucleus. Glycoconj J.

[49] Vyakarnam A, Dagher SF, Wang JL, Patterson RJ (1997). Evidence for a role for galectin-1 in pre-mRNA splicing. Mol Cell Biol.

[50] Schwarz FP, Ahmed H, Bianchet MA, Amzel LM, Vasta GR (1998). Thermodynamics of bovine spleen galectin-1 binding to disaccharides: correlation with structure and its effect on oligomerization at the denaturation temperature. Biochemistry.

[51] van der Leij J, van den Berg A, Blokzijl T (2004). Dimeric galectin-1 induces IL-10 production in T-lymphocytes: an important tool in the regulation of the immune response. J Pathol.

[52] Femel J, van Hooren L, Herre M (2022). Vaccination against galectin-1 promotes cytotoxic T-cell infiltration in melanoma and reduces tumor burden. Cancer Immunol Immunother.

[53] Dalotto-Moreno T, Croci DO, Cerliani JP (2013). Targeting galectin-1 overcomes breast cancer-associated immunosuppression and prevents metastatic disease. Cancer Res.

[54] Nambiar DK, Viswanathan V, Cao H (2023). Galectin-1 mediates chronic STING activation in tumors to promote metastasis through MDSC recruitment. Cancer Res.

[55] Mileo AM, Miccadei S (2016). Polyphenols as modulator of oxidative stress in cancer disease: new therapeutic strategies. Oxid Med Cell Longev.

[56] Gorrini C, Harris IS, Mak TW (2013). Modulation of oxidative stress as an anticancer strategy. Nat Rev Drug Discov.

[57] DeNicola GM, Karreth FA, Humpton TJ (2011). Oncogene-induced Nrf2 transcription promotes ROS detoxification and tumorigenesis. Nature.

[58] Ramos-Gomez M, Kwak MK, Dolan PM (2001). Sensitivity to carcinogenesis is increased and chemoprotective efficacy of enzyme inducers is lost in *nrf2* transcription factor-deficient mice. Proc Natl Acad Sci U S A.

[59] Hayes JD, McMahon M (2006). The double-edged sword of Nrf2: subversion of redox homeostasis during the evolution of cancer. Mol Cell.

[60] Tossetta G, Fantone S, Montanari E, Marzioni D, Goteri G (2022). Role of NRF2 in ovarian cancer. Antioxidants.

[61] Staurengo-Ferrari L, Badaro-Garcia S, Hohmann MSN (2018). Contribution of Nrf2 modulation to the mechanism of action of analgesic and anti-inflammatory drugs in pre-clinical and clinical stages. Front Pharmacol.

[62] Bao L, Wu J, Dodson M (2017). *ABCF2*, an Nrf2 target gene, contributes to cisplatin resistance in ovarian cancer cells. Mol Carcinog.

[63] Campagna R, Pozzi V, Giorgini S (2023). Paraoxonase-2 is upregulated in triple negative breast cancer and contributes to tumor progression and chemoresistance. Hum Cell.

[64] Campagna R, Bacchetti T, Salvolini E (2020). Paraoxonase-2 silencing enhances sensitivity of A375 melanoma cells to treatment with cisplatin. Antioxidants.

[65] Campagna R, Belloni A, Pozzi V (2022). Role played by paraoxonase-2 enzyme in cell viability, proliferation and sensitivity to chemotherapy of oral squamous cell carcinoma cell lines. Int J Mol Sci.

[66] Fumarola S, Cecati M, Sartini D (2020). Bladder cancer chemosensitivity is affected by paraoxonase-2 expression. Antioxidants.

[67] Nagarajan A, Dogra SK, Sun L (2017). Paraoxonase 2 facilitates pancreatic cancer growth and metastasis by stimulating GLUT1-mediated glucose transport. Mol Cell.

[68] Tseng JH, Chen CY, Chen PC (2017). Valproic acid inhibits glioblastoma multiforme cell growth via paraoxonase 2 expression. Oncotarget.

[69] Barrera G, Cucci MA, Grattarola M, Dianzani C, Muzio G, Pizzimenti S (2021). Control of oxidative stress in cancer chemoresistance: spotlight on Nrf2 role. Antioxidants.

[70] Sferrazzo G, Di Rosa M, Barone E (2020). Heme oxygenase-1 in central nervous system malignancies. J Clin Med.

[71] Niture SK, Jaiswal AK (2013). Nrf2-induced antiapoptotic Bcl-xL protein enhances cell survival and drug resistance. Free Radic Biol Med.

[72] Saha S, Buttari B, Panieri E, Profumo E, Saso L (2020). An overview of Nrf2 signaling pathway and its role in inflammation. Molecules.

[73] Kansanen E, Kivelä AM, Levonen AL (2009). Regulation of Nrf2-dependent gene expression by 15-deoxy-Δ^12,14^-prostaglandin J_2_. Free Radic Biol Med.

[74] He F, Ru X, Wen T (2020). NRF2, a transcription factor for stress response and beyond. Int J Mol Sci.

[75] Itoh K, Wakabayashi N, Katoh Y (1999). Keap1 represses nuclear activation of antioxidant responsive elements by Nrf2 through binding to the amino-terminal Neh2 domain. Genes Dev.

[76] Panieri E, Saso L (2019). Potential applications of NRF2 inhibitors in cancer therapy. Oxid Med Cell Longev.

[77] Sova M, Saso L (2018). Design and development of Nrf2 modulators for cancer chemoprevention and therapy: a review. Drug Des Devel Ther.

[78] Dinkova-Kostova AT, Kostov RV, Canning P (2017). Keap1, the cysteine-based mammalian intracellular sensor for electrophiles and oxidants. Arch Biochem Biophys.

[79] Taguchi K, Motohashi H, Yamamoto M (2011). Molecular mechanisms of the Keap1-Nrf2 pathway in stress response and cancer evolution. Genes Cells.

[80] Kansanen E, Kuosmanen SM, Leinonen H, Levonen AL (2013). The Keap1-Nrf2 pathway: mechanisms of activation and dysregulation in cancer. Redox Biol.

[81] Li W, Yu S, Liu T (2008). Heterodimerization with small Maf proteins enhances nuclear retention of Nrf2 via masking the NESzip motif. Biochim Biophys Acta.

[82] Itoh K, Chiba T, Takahashi S (1997). An Nrf2/small Maf heterodimer mediates the induction of phase II detoxifying enzyme genes through antioxidant response elements. Biochem Biophys Res Commun.

[83] Bayo Jimenez MT, Frenis K, Hahad O (2022). Protective actions of nuclear factor erythroid 2-related factor 2 (NRF2) and downstream pathways against environmental stressors. Free Radic Biol Med.

[84] Cuadrado A, Manda G, Hassan A (2018). Transcription factor NRF2 as a therapeutic target for chronic diseases: a systems medicine approach. Pharmacol Rev.

[85] Kaspar JW, Niture SK, Jaiswal AK (2009). Nrf2:INrf2 (Keap1) signaling in oxidative stress. Free Radic Biol Med.

[86] Shaw P, Chattopadhyay A (2020). Nrf2-ARE signaling in cellular protection: mechanism of action and the regulatory mechanisms. J Cell Physiol.

[87] Stefanson AL, Bakovic M (2014). Dietary regulation of Keap1/Nrf2/ARE pathway: focus on plant-derived compounds and trace minerals. Nutrients.

[88] Choi AM, Alam J (1996). Heme oxygenase-1: function, regulation, and implication of a novel stress-inducible protein in oxidant-induced lung injury. Am J Respir Cell Mol Biol.

[89] Sun J, Brand M, Zenke Y, Tashiro S, Groudine M, Igarashi K (2004). Heme regulates the dynamic exchange of Bach1 and NF-E2-related factors in the Maf transcription factor network. Proc Natl Acad Sci U S A.

[90] Kaspar JW, Jaiswal AK (2010). Antioxidant-induced phosphorylation of tyrosine 486 leads to rapid nuclear export of Bach1 that allows Nrf2 to bind to the antioxidant response element and activate defensive gene expression. J Biol Chem.

[91] Zenke-Kawasaki Y, Dohi Y, Katoh Y (2007). Heme induces ubiquitination and degradation of the transcription factor Bach1. Mol Cell Biol.

[92] Lignitto L, LeBoeuf SE, Homer H (2019). Nrf2 activation promotes lung cancer metastasis by inhibiting the degradation of Bach1. Cell.

[93] Wiel C, Le Gal K, Ibrahim MX (2019). BACH1 stabilization by antioxidants stimulates lung cancer metastasis. Cell.

[94] Gall Trošelj K, Tomljanović M, Jaganjac M (2022). Oxidative stress and cancer heterogeneity orchestrate NRF2 roles relevant for therapy response. Molecules.

[95] (2018). Rojo de la Vega M, Chapman E, Zhang DD. NRF2 and the hallmarks of cancer. Cancer Cell.

[96] Iida K, Itoh K, Kumagai Y (2004). Nrf2 is essential for the chemopreventive efficacy of oltipraz against urinary bladder carcinogenesis. Cancer Res.

[97] Mitsuishi Y, Taguchi K, Kawatani Y (2012). Nrf2 redirects glucose and glutamine into anabolic pathways in metabolic reprogramming. Cancer Cell.

[98] Sporn MB, Liby KT (2012). NRF2 and cancer: the good, the bad and the importance of context. Nat Rev Cancer.

[99] Rubinstein N, Alvarez M, Zwirner NW (2004). Targeted inhibition of galectin-1 gene expression in tumor cells results in heightened T cell-mediated rejection; a potential mechanism of tumor-immune privilege. Cancer Cell.

[100] Liu FT, Rabinovich GA (2005). Galectins as modulators of tumour progression. Nat Rev Cancer.

[101] Tang D, Yuan Z, Xue X (2012). High expression of *Galectin-1* in pancreatic stellate cells plays a role in the development and maintenance of an immunosuppressive microenvironment in pancreatic cancer. Int J Cancer.

[102] Tsai YT, Liang CH, Yu JH (2019). A DNA aptamer targeting galectin-1 as a novel immunotherapeutic strategy for lung cancer. Mol Ther Nucleic Acids.

[103] Perillo NL, Pace KE, Seilhamer JJ, Baum LG (1995). Apoptosis of T cells mediated by galectin-1. Nature.

[104] Górniak P, Wasylecka-Juszczyńska M, Ługowska I (2020). BRAF inhibition curtails IFN-gamma-inducible PD-L1 expression and upregulates the immunoregulatory protein galectin-1 in melanoma cells. Mol Oncol.

[105] You Y, Tan JX, Dai HS (2016). MiRNA-22 inhibits oncogene galectin-1 in hepatocellular carcinoma. Oncotarget.

[106] Soldati R, Berger E, Zenclussen AC (2012). Neuroblastoma triggers an immunoevasive program involving galectin-1-dependent modulation of T cell and dendritic cell compartments. Int J Cancer.

[107] Huang EY, Chen YF, Chen YM (2012). A novel radioresistant mechanism of galectin-1 mediated by H-Ras-dependent pathways in cervical cancer cells. Cell Death Dis.

[108] Chung LY, Tang SJ, Sun GH (2012). Galectin-1 promotes lung cancer progression and chemoresistance by upregulating p38 MAPK, ERK, and cyclooxygenase-2. Clin Cancer Res.

[109] Carabias P, Espelt MV, Bacigalupo ML (2022). Galectin-1 confers resistance to doxorubicin in hepatocellular carcinoma cells through modulation of P-glycoprotein expression. Cell Death Dis.

[110] Bacigalupo ML, Manzi M, Espelt MV (2015). Galectin-1 triggers epithelial-mesenchymal transition in human hepatocellular carcinoma cells. J Cell Physiol.

[111] Nam K, Son SH, Oh S (2017). Binding of galectin-1 to integrin β1 potentiates drug resistance by promoting survivin expression in breast cancer cells. Oncotarget.

[112] Wang F, Lv P, Gu Y, Li L, Ge X, Guo G (2017). Galectin-1 knockdown improves drug sensitivity of breast cancer by reducing P-glycoprotein expression through inhibiting the Raf-1/AP-1 signaling pathway. Oncotarget.

[113] Le Mercier M, Mathieu V, Haibe-Kains B (2008). Knocking down galectin 1 in human hs683 glioblastoma cells impairs both angiogenesis and endoplasmic reticulum stress responses. J Neuropathol Exp Neurol.

[114] Ito K, Scott SA, Cutler S (2011). Thiodigalactoside inhibits murine cancers by concurrently blocking effects of galectin-1 on immune dysregulation, angiogenesis and protection against oxidative stress. Angiogenesis.

[115] Pizzino G, Irrera N, Cucinotta M (2017). Oxidative stress: harms and benefits for human health. Oxid Med Cell Longev.

[116] Raffaghello L, Lee C, Safdie FM (2008). Starvation-dependent differential stress resistance protects normal but not cancer cells against high-dose chemotherapy. Proc Natl Acad Sci U S A.

[117] Pateras IS, Williams C, Gianniou DD (2023). Short term starvation potentiates the efficacy of chemotherapy in triple negative breast cancer via metabolic reprogramming. J Transl Med.

[118] Hogas S, Bilha SC, Branisteanu D (2017). Potential novel biomarkers of cardiovascular dysfunction and disease: cardiotrophin-1, adipokines and galectin-3. Arch Med Sci.

[119] Reuter S, Gupta SC, Chaturvedi MM, Aggarwal BB (2010). Oxidative stress, inflammation, and cancer: how are they linked?. Free Radic Biol Med.

[120] Tazhitdinova R, Timoshenko AV (2020). The emerging role of galectins and O-GlcNAc homeostasis in processes of cellular differentiation. Cells.

[121] Matarrese P, Tinari A, Mormone E (2005). Galectin-1 sensitizes resting human T lymphocytes to Fas (CD95)-mediated cell death via mitochondrial hyperpolarization, budding, and fission. J Biol Chem.

[122] Thijssen VL, Postel R, Brandwijk RJ (2006). Galectin-1 is essential in tumor angiogenesis and is a target for antiangiogenesis therapy. Proc Natl Acad Sci U S A.

[123] Wada J, Makino H (2001). Galectins, galactoside-binding mammalian lectins: clinical application of multi-functional proteins. Acta Med Okayama.

[124] Weng IC, Chen HL, Lo TH (2018). Cytosolic galectin-3 and -8 regulate antibacterial autophagy through differential recognition of host glycans on damaged phagosomes. Glycobiology.

[125] Karlsson A, Follin P, Leffler H, Dahlgren C (1998). Galectin-3 activates the NADPH-oxidase in exudated but not peripheral blood neutrophils. Blood.

[126] Wolf Y, Anderson AC, Kuchroo VK (2020). TIM3 comes of age as an inhibitory receptor. Nat Rev Immunol.

[127] Solinas C, De Silva P, Bron D, Willard-Gallo K, Sangiolo D (2019). Significance of TIM3 expression in cancer: from biology to the clinic. Semin Oncol.

[128] Rezaei M, Ghanadian M, Ghezelbash B (2023). TIM-3/Gal-9 interaction affects glucose and lipid metabolism in acute myeloid leukemia cell lines. Front Immunol.

[129] Shen L, Lu K, Chen Z, Zhu Y, Zhang C, Zhang L (2023). Pre-treatment with galectin-1 attenuates lipopolysaccharide-induced myocarditis by regulating the Nrf2 pathway. Eur J Histochem.

[130] Liu HB, Li QY, Zhang XD, Shi Y, Li JY (2022). The neuroprotective effects of Galectin-1 on Parkinson’s disease via regulation of Nrf2 expression. Eur Rev Med Pharmacol Sci.

[131] Park SY, Chung YS, Park SY, Kim SH (2022). Role of AMPK in regulation of oxaliplatin-resistant human colorectal cancer. Biomedicines.

[132] Petsouki E, Cabrera SNS, Heiss EH (2022). AMPK and NRF2: interactive players in the same team for cellular homeostasis?. Free Radic Biol Med.

[133] Huang XT, Liu W, Zhou Y (2020). Galectin-1 ameliorates lipopolysaccharide-induced acute lung injury via AMPK-Nrf2 pathway in mice. Free Radic Biol Med.

[134] Wells V, Mallucci L (1991). Identification of an autocrine negative growth factor: mouse beta-galactoside-binding protein is a cytostatic factor and cell growth regulator. Cell.

[135] Astorgues-Xerri L, Riveiro ME, Tijeras-Raballand A (2014). OTX008, a selective small-molecule inhibitor of galectin-1, downregulates cancer cell proliferation, invasion and tumour angiogenesis. Eur J Cancer.

[136] Zucchetti M, Bonezzi K, Frapolli R (2013). Pharmacokinetics and antineoplastic activity of galectin-1-targeting OTX008 in combination with sunitinib. Cancer Chemother Pharmacol.

[137] Dings RP, Kumar N, Miller MC (2013). Structure-based optimization of angiostatic agent 6DBF7, an allosteric antagonist of galectin-1. J Pharmacol Exp Ther.

[138] Croci DO, Salatino M, Rubinstein N (2012). Disrupting galectin-1 interactions with N-glycans suppresses hypoxia-driven angiogenesis and tumorigenesis in Kaposi’s sarcoma. J Exp Med.

